# Mean bond-length variations in crystals for ions bonded to oxygen

**DOI:** 10.1107/S2052520617014548

**Published:** 2017-11-28

**Authors:** Olivier Charles Gagné, Frank Christopher Hawthorne

**Affiliations:** a Geological Sciences, University of Manitoba, 125 Dysart Road, Winnipeg, Manitoba R3T 2N2, Canada

**Keywords:** bond length, coordination number, distortion, electronegativity, ionization energy, structural strain, ionic radius, oxide, oxysalt

## Abstract

Variations in mean bond length are examined in oxide and oxysalt crystals for 55 cation configurations bonded to O^2−^. Stepwise multiple regression analysis shows that mean bond length is correlated to bond-length distortion in 42 ion configurations at the 95% confidence level, with a mean coefficient of determination (〈*R* ^2^〉) of 0.35. Previously published correlations between mean bond length and mean coordination number of the bonded anions are found not to be of general applicability to inorganic oxide and oxysalt structures. For two of 11 ions tested for the 95% confidence level, mean bond lengths predicted using a fixed radius for O^2−^ are significantly more accurate as those predicted using an O^2−^ radius dependent on coordination number, and are statistically identical otherwise. As a result, the currently accepted ionic radii for O^2−^ in different coordinations are not justified by experimental data. Previously reported correlation between mean bond length and the mean electronegativity of the cations bonded to the oxygen atoms of the coordination polyhedron is shown to be statistically insignificant; similar results are obtained with regard to ionization energy. It is shown that *a priori* bond lengths calculated for many ion configurations in a single structure-type leads to a high correlation between *a priori* and observed mean bond lengths, but *a priori* bond lengths calculated for a single ion configuration in many different structure-types leads to negligible correlation between *a priori* and observed mean bond lengths. This indicates that structure type has a major effect on mean bond length, the magnitude of which goes beyond that of the other variables analyzed here.

## Abstract 2

Variations in mean bond length are examined in oxide and oxysalt crystals for 55 cation configurations bonded to O^2−^. Bond-length distortion is confirmed as a statistically significant causal factor of mean bond-length variation. The assignment of a coordination-based radius to O^2−^ is found not to be supported by experimental data.

## 1. Introduction   

In the 1960s and 1970s, a considerable amount of work was carried out on trying to understand the reasons underlying variations in mean bond length in crystals. This resulted from the improving precision of structure refinements which began showing variations in mean bond length that significantly exceeded experimental error. Several factors were examined as possible sources of this variation, and many studies were reported as ‘reasonably successful’ in correlating variation in mean bond length with one or more possible causal factors, *e.g.* variation in mean coordination number of the bonded anions, variation in mean electronegativity of the next-nearest-neighbour cations, dispersion of bond lengths about their mean value (distortion). However, these studies were typically limited to a single configuration of the oxidation state and coordination number of an ion, and often consisted of few data.

### 1.1. Anion coordination number   

Smith & Bailey (1963 ▸) examined bond lengths for Si^4+^O_4_ and Al^3+^O_4_ tetrahedra in a series of silicate and alumino­silicate minerals and attributed variation in mean bond length to the degree of polymerization of these tetrahedra. Shannon & Prewitt (1969 ▸) provided the first list of ionic radii as a function of coordination number for anions, citing the influence of the work of Goldschmidt *et al.* (1926 ▸) and Slaughter (1966 ▸), and went on to propose that the variation observed by Smith & Bailey (1963 ▸) was actually due to variation in mean anion-coordination number (〈CN〉). Brown & Gibbs (1969 ▸) correlated mean bond length to mean anion coordination for SiO_4_ in 46 structures, reporting a coefficient of determination (*R* ^2^) of 0.6. However, their data set excluded feldspars and zeolites due to the ambiguity of the anion coordination number, and several sodium silicates, citing the development of strong *d*–*p* π-bonding in the presence of highly electropositive Na that seemed to override the effect of cation coordination of O^2−^ on the Si—O bond (now discredited). While recognizing appreciable discrepancy in different structure types, Baur (1971 ▸) commented that ‘this correlation is without doubt valid’, but noted that the discrepancy between observed and calculated mean Si—O distances is appreciable in certain structures, *e.g.* 0.017 Å in topaz. Since the beginning of the 1970s, it has been accepted that the radius of O^2−^ varies monotonically as a function of its coordination number.

### 1.2. Electronegativity   

Following proposals by Noll (1963 ▸) and Lazarev (1964 ▸) that *individual* interatomic distances depend on the electronegativity of nearest-neighbour cations in the structure, Brown & Gibbs (1969 ▸, 1970 ▸) showed a correlation between individual bond lengths and (1) electronegativity of the non-tetrahedrally coordinated cations in four isostructural *C*2/*m* amphiboles (tremolite, Mn-cummingtonite, glaucophane and grunerite), (2) the residual electron density on O, and (3) bond order. Shannon (1971 ▸) found a positive correlation between effective ionic radius (mean bond length corrected for anion coordination number) and the average electronegativity of all cations in the structure for *T*O_4_ polyhedra (*T* = B^3+^, Si^4+^, P^5+^, As^5+^, V^5+^, S^6+^, Se^6+^, Cr^6+^ and Mo^6+^). Baur (1971 ▸) examined a series of strictly isostructural pyroxenes with the formula *MM*′Si_2_O_6_ and found no correlation of individual ^[4]^Si^4+^—O distances with the electronegativity of the *M* ion. He states that a rigorous examination of *C*2/*m* amphiboles was not possible due to wide variation in anion coordination number and valence state of the next-nearest-neighbour cations, making the different amphiboles not strictly isostructural. Baur concludes that the search for a correlation between bond length and the electronegativity of the other cations in the structure is probably futile for ^[4]^Si^4+^—O bonds.

Shannon & Calvo (1973*a* ▸) analyzed *mean* bond lengths in 62 phosphates, 21 arsenates and 22 vanadates and showed correlations between the observed mean bond lengths and the mean electronegativity of the cations in the structure (*R* ^2^ = 0.01–0.30), and between the effective ionic radius (the mean bond length minus the radius of O^2−^ as a function of coordination number) and the mean electronegativity of the cations in the structure (*R* ^2^ = 0.46–0.72). They list other potential factors affecting mean bond lengths worth considering, namely the valence of the next-nearest-neighbour cations and their bond lengths to oxygen. Jeitschko *et al.* (1976 ▸) also found correlations (*R* ^2^ not given) between mean ^[4]^Fe^3+^—O bond lengths and the electronegativity of the next-nearest neighbour cations and the ^57^Fe Mössbauer isomer shift for six oxide structures.

### 1.3. Bond-length distortion   

Brown & Shannon (1973 ▸) correlated mean bond length with the mean-square relative deviation of bond lengths from their average value (herein referred to as *distortion*, Δ) for V^5+^, Cu^2+^, Mg^2+^, Li^+^, Zn^2+^ and Co^2+^ in octahedral coordination (sample sizes ∼ 20 coordination polyhedra), reporting *R* ^2^ values ranging from 0.18 to 0.96, where
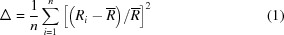
for a coordination number *n*. They point out that the correlation is remarkably high for ions showing large distortion, and that other effects such as anion coordination become more important in explaining mean bond-length variations in slightly distorted octahedra. Shannon & Calvo (1973*b* ▸) and Shannon *et al.* (1975 ▸) showed similar correlations for ^[6]^Cu^2+^—O and ^[6]^Mn^3+^—O, respectively, with *R* ^2^ = 0.79 for 25 Cu^2+^—O octahedra and *R* ^2^ = 0.67 for 16 Mn^3+^—O octahedra in 25 crystal structures. Shannon (1976 ▸) showed the dependence of mean bond length on distortion for a series of ion configurations octahedrally coordinated to O^2−^: Mo^6+^ (*n* = 38, *R* ^2^ = 0.55), W^6+^ (*n* = 7, *R* ^2^ = 0.56), V^5+^ (*n* = 16, *R* ^2^ = 0.96), Nb^5+^(*n* = 29, *R* ^2^ = 0.48), Ta^5+^ (*n* = 6, *R* ^2^ = 0.66), Mn^3+^ (*n* = 15, *R* ^2^ = 0.67), Cu^2+^ (*n* = 26, *R* ^2^ = 0.67), Mg^2+^ (*n* = 28, *R* ^2^ = 0.52), Co^2+^ (*n* = 15, *R* ^2^ = 0.18), Zn^2+^ (*n* = 16, *R* ^2^ = 0.41) and Li^+^ (*n* = 11, *R* ^2^ = 0.66). Shannon (1976 ▸) cites partial site occupancy, covalence (*i.e.* differences in electronegativity of the bonded atoms) and electron delocalization (in non-oxides) as other potential factors affecting mean bond length. Baur (1974 ▸) showed that mean ^[4]^P^5+^—O bond lengths are affected by both anion coordination and tetrahedral distortion for a sample of 211 phosphate tetrahedra: *R* ^2^ = 0.03 for dependence on anion 〈CN〉 (*R* ^2^ = 0.24 when correcting for distortion), and *R* ^2^ = 0.12 for dependence on distortion (*R* ^2^ = 0.36 when correcting for anion 〈CN〉). Baur found no correlation between mean bond length and O—O distance in phosphates, and stated that additional factors must be at play in explaining mean bond-length variations, citing the average electronegativity of the cations bonded to the oxygen atoms as a potential candidate.

Baur (1977 ▸, 1978 ▸) examined the correlation of anion coordination number, average cation electronegativity in the structure, tetrahedral distortion and other potential factors on mean bond length for 314 ^[4]^Si^4+^—O polyhedra, and with a multiple regression analysis showed that only anion coordination number, the number of bridging anions per tetrahedron and the mean value of the secant of the bridging angles Si—O—*T* correlate significantly with mean bond-length variations (combined *R* ^2^ = 0.58). Electronegativity and distortion showed no correlation to mean bond length. Baur (1978 ▸) assessed the significance of the correlations between ^[4]^Si^4+^—O mean bond-length and the variables he studied *via t*-tests (presumably from the multiple regression and not from individual correlations). His statistical analysis was more rigorous than similar studies at the time; however, Baur mentions (1) correlation between some of the independent variables he used, and stated that he tried to minimize their effect and (2) reports a *combined R* ^2^ value of 0.58 between mean bond length and the anion coordination number and the number of bridging anions per tetrahedron, and 0.65 to the secant of the bridging angles Si—O—*T*. Stepwise regression analysis and the reporting of *adjusted R* ^2^ values would have further improved the validity of the results.

Hawthorne & Faggiani (1979 ▸) used 41 ^[4]^V^5+^—O coordination polyhedra to show a correlation (*R* ^2^ not reported) between mean bond length and anion coordination number and the average electronegativity of the next-nearest-neighbour cations (as opposed to the electronegativity of *all* cations in the structure, as was carried out before that), finding no significant correlation with tetrahedral distortion, tetrahedral angle variance or the number of bridging anions per tetrahedron.

Much has since been written about bond-length distortion and its effect on mean bond length, focusing particularly on the bond-valence model of chemical bonding (*e.g.* Brown, 1992 ▸, 2002 ▸, 2006 ▸, 2009 ▸, 2014 ▸, 2016 ▸; Urusov & Orlov, 1999 ▸; Urusov, 2003 ▸, 2006*b* ▸, 2014 ▸; Bosi, 2014 ▸). Hawthorne *et al.* (1996 ▸) found no significant correlation between mean bond length and distortion for BO_3_ and BO_4_ groups. Urusov (2006*a* ▸) examined mean bond-length variations in Mn^3+^F_6_ octahedra and described a correlation with distortion with *R* ^2^ = 0.38 for 116 coordination polyhedra. Urusov (2008 ▸) described a correlation of mean bond length and distortion for Mo^6+^O_6_ octahedra, with *R* ^2^ = 0.28 in 826 coordination polyhedra, and Urusov & Serezhkin (2009 ▸) reported *R* ^2^ values of 0.83 and 0.46 for 190 V^5+^O_6_ polyhedra and 200 V^4+^O_6_ polyhedra, respectively, as well as a qualitative correlation for V^3+^O_6_ and V^2+^O_6_.

More recently, Gagné & Hawthorne (2016*a* ▸) showed a correlation between mean bond length and (1) bond-length distortion and (2) the ratio of *U*_eq_ or *B* (the mean atom displacement derived during crystal structure refinement) between ^[6]^Na^+^ and its bonded anions in oxides, with *R* ^2^ = 0.52 for distortion, 0.57 for *U*_eq_ ratio, and an adjusted *R* ^2^ of 0.68 for both (*n* = 56), and showed for alkali-metal and alkaline-earth-metal ions bonded to O^2−^ that the extent of distortion is highly correlated to the observed curvature of the bond-valence curve of the isoelectronic series to which the constituent ion belongs.

## 2. Purpose of this work   

Persuasive examples have been reported describing correlations between mean bond length and bond-length distortion for ion configurations prone to large distortions (*e.g.* Brown & Shannon, 1973 ▸; Shannon, 1976 ▸) but the generality of this relation is not established for all ion configurations. Furthermore, the importance of other factors outlined above remains unclear. Here, we clarify these effects by using the results of a very large bond-length dispersion analysis carried out by the authors for 135 cations in 462 configurations, for a total of 180 331 bond lengths and 31 514 coordination polyhedra from 9367 refined crystal structures (described by Gagné & Hawthorne, 2015 ▸, 2016*a* ▸, 2017*a* ▸,*b* ▸; Gagné, 2017 ▸). Our data set is being released as it is published (*e.g.* Gagné & Hawthorne, 2016*a* ▸) with the hope of encouraging further detailed studies.

## 3. Variables considered in this work   

The following variables were systematically evaluated for all ion configurations as potential causal factors underlying mean bond-length variation: (1) bond-length distortion, (2) mean coordination number of the oxygen atoms bonded to the cation (previous references used the less accurate mean coordination number of *all* oxygen atoms of the structure), (3) mean electronegativity (〈χ〉) of the cations bonded to the oxygen atoms of the coordination polyhedron, (4) mean ionization energy (〈*IE*〉) of the cations bonded to the oxygen atoms of the coordination polyhedron (new).

Other factors can affect observed mean bond lengths, *e.g.* multiple occupancy of a site, partial site occupancy, electron delocalization, but these effects have (hopefully) been eliminated from our data set by careful filtering. Other potential errors may be present in the data and lead to unaccounted variability in mean bond length: undetected errors in the ICSD database (*e.g.* transcription), experimental variability/uncertainty, experimental error, and failure to report solid solution at the site of interest.

## 4. Statistical significance   

The reporting of statistical significance for the correlations cited in the *Introduction* [Sec sec1] has been scarce. This is a cause for concern as some of the correlations reported as ‘significant’ have not been tested for a specific confidence level. Here, we have tested all correlations for statistical significance on an individual basis, and *via* multiple-regression analysis (Student *t*-test, 95% confidence level). In addition, we have not excluded different types of data (*e.g.* alkali-metal silicates from a consideration of Si^4+^—O bonds) from our analysis in order to avoid bias due to pre-conceived notions of causality.

There are two distinct issues with regard to correlation: (1) whether the dependent parameter is significantly correlated to the independent parameter, which is determined from the *p*-value for the null hypothesis that the slope of the correlation between variables is equal to zero; (2) the value of *R* ^2^, which indicates the proportion of the variation of the dependent parameter that can be attributed to the independent variable. In particular, a correlation may be very low (*i.e.* a small *R* ^2^ value), but the dependent parameter can nonetheless be significantly correlated to the independent parameter (*i.e. p* < 0.05 and 0.01 for the 95 and 99% confidence levels, respectively).

## 5. The effect of sample size   

In order to gauge the reliability of the correlations developed here, we have examined the variation of (1) *p*-values, and (2) *R* ^2^ values as a function of sample size for SiO_4_, using data taken at random from our data set of 334 coordination polyhedra. For a large set of data, one expects *p*-values to be independent of sample size, such that a statistically significant correlation may be ascribed as such regardless of the number of data. As the number of data decreases, eventually the *p*-values and *R* ^2^ values will become unreliable, and it is obviously important to know at what number of data the analysis begins to become unreliable.

**Figure 1:**
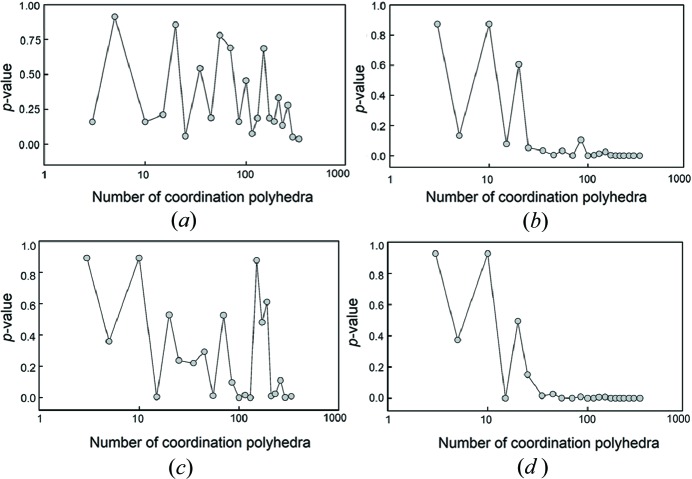
The effect of sample size on the statistical significance of the correlation between mean bond length and (*a*) bond-length distortion, (*b*) mean coordination number of the oxygen atoms bonded to the cation, (*c*) mean electronegativity and (*d*) mean ionization energy of the cations bonded to the oxygen atoms of the coordination polyhedron, as measured by *p*-values, for SiO_4_.

**Figure 2:**
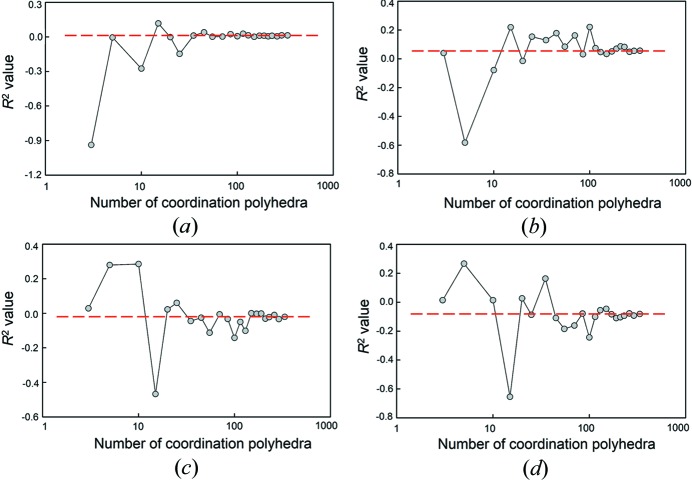
The effect of sample size on *R* ^2^ for (*a*) bond-length distortion, (*b*) mean coordination number of the oxygen atoms bonded to the cation, (*c*) mean electronegativity and (*d*) mean ionization energy of the cations bonded to the oxygen atoms of the coordination polyhedron, for SiO_4_. The dashed lines show the value for the parent distribution (*n* = 334).

Fig. 1 ▸ shows the effect of sample size on the statistical significance of the correlation between mean bond length and (*a*) bond-length distortion, (*b*) mean coordination number of the oxygen atoms bonded to the cation, (*c*) mean electronegativity, and (*d*) mean ionization energy of the cations bonded to the oxygen atoms of the coordination polyhedron, using *p*-values (*i.e.* the lower the *p*-value, the greater the significance of the correlation). Fig. 2 ▸ shows the corresponding effect on *R* ^2^. For distortion [Figs. 1(*a*) and 2(*a*)], the *p*-values are very irregular but are always > 0.05, indicating no significant correlation, but the *R* ^2^ values (which are ∼ 0.00 in the parent distribution) become irregular at lower numbers of data, and can show high correlations even though the *p*-values indicate that we may not reject the hypothesis that there is no correlation. For mean coordination number of the oxygen atoms bonded to the cation [Figs. 1(*b*) and 2(*b*)] and for mean ionization energy of the cations bonded to the oxygen atoms of the coordination polyhedron [Figs. 1(*d*) and 2(*d*)], the *p*-values are close to zero at larger numbers of data (indicative of significant correlation) and show low variability in *R* ^2^ (indicating the same degree of contribution to the variation in the dependent variable), but both *p*-values and *R* ^2^ values oscillate wildly for small numbers of data. The behaviour for the mean electronegativity of the cations bonded to the oxygen atoms resembles that of distortion except that for some number of samples, the *p*-values suggest significant correlation although most of the corresponding *R* ^2^ values do not. To summarize, Figs. 1 ▸ and 2 ▸ indicate a strong drop in reliability of the regression-analysis results below ∼ 35 coordination polyhedra, and suggest that robust results require at least 100 coordination polyhedra. A notable accidental correlation found from this study of sample size is that of mean bond length and mean ionization energy of the cations bonded to the oxygen atoms of the coordination polyhedron, which is found to be statistically significant at the 99.9% confidence level, with *R* ^2^ = −0.66 for 15 polyhedra [Figs. 1(*d*) and 2(*d*)], whereas *R* ^2^ = 0.08 for the parent distribution with *n* = 334 (note that throughout this work, a negative symbol before *R* ^2^ indicates that the observed correlation with mean bond length is negative).

Here, we report correlations for sample sizes as low as 16 coordination polyhedra, primarily to make these data available to people working on these compositions, but note that conclusions drawn for ion configurations with less than ∼ 100 coordination polyhedra cannot be considered statistically reliable; this proviso extends to conclusions drawn from previous studies.

## 6. Stepwise regression analysis   

A stepwise regression analysis based on *t*-tests (95% confidence level) was used to eliminate misleading correlations between the variables of this study. A step-by-step procedure of the stepwise regression analysis for ^[6]^Na^+^ is shown in Table 1 ▸. When individually correlated to mean bond length, *p*-values for (1) bond-length distortion, (2) mean coordination of the bonded anions, (3) mean electronegativity (〈χ〉)and (4) mean ionization energy (〈*IE*〉) of the next-nearest-neighbour cations are 5.4 × 10^−8^, 1.9 × 10^−3^, 1.5 × 10^−3^, 0.26, respectively. The first step of the regression analysis shows that factoring in the variable with lowest *p*-value (italicized, *i.e.* distortion), leads to a *R* ^2^ value of 0.24. The *p*-values of the other three variables update, and the mean electronegativity of the next-nearest-neighbour cation then gets factored in as the remaining variable with the lowest *p*-value below the threshold of 0.05, leading to an adjusted *R* ^2^ value of 0.27. Interestingly, this then results in a *p*-value of 0.24 (above 0.05) for the mean coordination of the bonded anions. However, including the mean ionization energy of the next-nearest-neighbour cation in the next step (its *p*-value having dropped below 0.05) brings back its *p*-value below 0.05 for the final step. Thus all variables get included in the regression analysis, and are construed as significant. We note that running the regression for a 99% confidence level, the refinement would have stopped after step 2, and only bond-length distortion and the mean electronegativity of the next-nearest-neighbour cation would have been included as significant.

Another example of correlation between variables was observed for ^[6]^V^5+^, for which individual *p*-values of 4.9 × 10^−4^ and 8.1 × 10^−4^ are obtained for the distortion and ionization energy, respectively, and 0.079 for electronegativity. Including either distortion or ionization energy in the first step of the multiple regression subsequently lowers the *p*-value for electronegativity below the threshold of 0.05, but brings the other variable above the threshold to ∼ 0.2–0.3 which is then discarded. In this situation, distortion is the variable factored in for having the lowest *p*-value. These issues show that correlation of individual variables to mean bond length can be misleading, and it is imperative that this effect be dealt with appropriately *via* stepwise regression analysis due to the less manageable issues we have raised above with regard to statistical significance and sample size.

## 7. Results   

We selected samples for 55 ion configurations from our bond-length dispersion analysis, bonded solely to O^2−^, with a minimum size of ∼ 20 coordination polyhedra. Stepwise regression analysis was performed (based on *p*-values < 0.05) with mean bond length as the dependent variable and (1) bond-length distortion, (2) mean coordination of the bonded anions, (3) mean electronegativity and (4) mean ionization energy of the next-nearest-neighbour cations as independent variables. Results are listed in Table 2 ▸ for correlations significant at the 95% confidence level (at 99% shown in bold) in the form of *R* ^2^ values, and adjusted *R* ^2^ values for ion configurations for which two or more potential factors are statistically significant for a 95% confidence level. Results are arranged in decreasing order of *R* ^2^. Of the 220 individual correlation trials (55 per variable), 77 are significant at the 95% confidence level and 62 at the 99% confidence level. Of the 77 correlations that are significant at the 99% confidence level, 42 involve distortion, 14 involve mean coordination of the bonded anions, 13 involve mean ionization energy of the next-nearest-neighbour cations, and eight involve the mean electronegativity of the next-nearest-neighbour cations.

### 7.1. Bond-length distortion   

The correlation between bond-length distortion and mean bond length is significant, and the underlying mechanism (the distortion theorem) is well known (*e.g.* Brown & Shannon, 1973 ▸; Allmann, 1975 ▸; Brown, 1978 ▸, 2002 ▸; Urusov, 2003 ▸).

**Figure 3:**
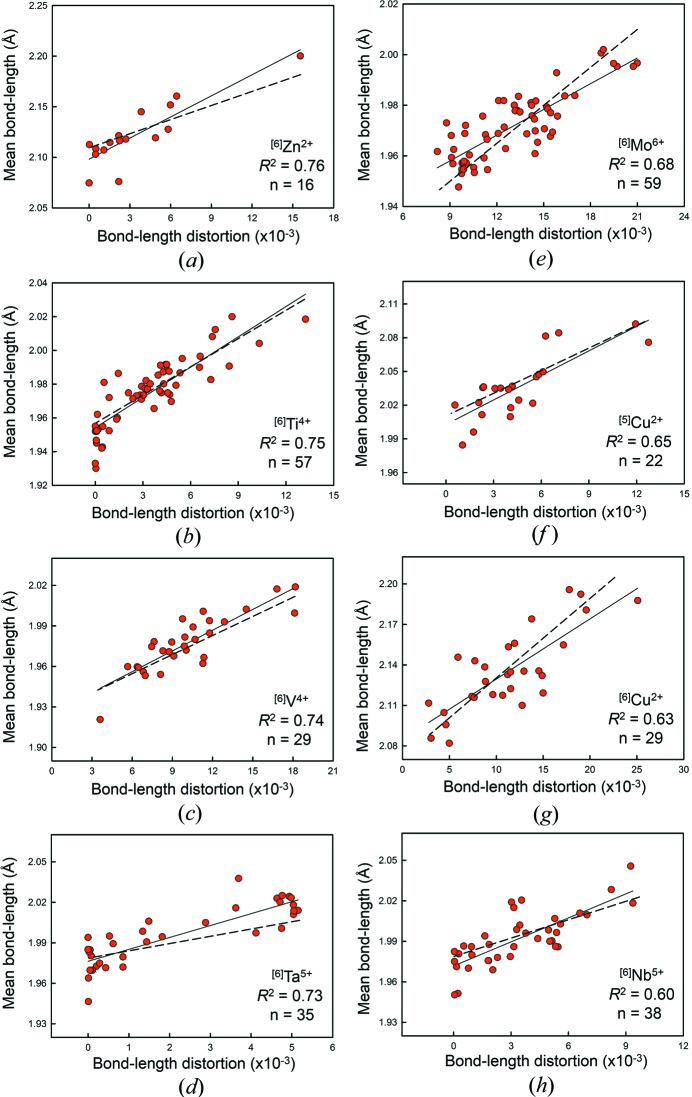
Bond-length distortion plots for (*a*) ^[6]^Zn^2+^, (*b*) ^[6]^Ti^4+^, (*c*) ^[6]^V^4+^, (*d*) ^[6]^Ta^5+^, (*e*) ^[6]^Mo^6+^, (*f*) ^[5]^Cu^2+^, (*g*) ^[6]^Cu^2+^ and (*h*) ^[6]^Nb^5+^ bonded to O^2−^. The dashed line indicates the equation predicted by the distortion theorem.

**Figure 4:**
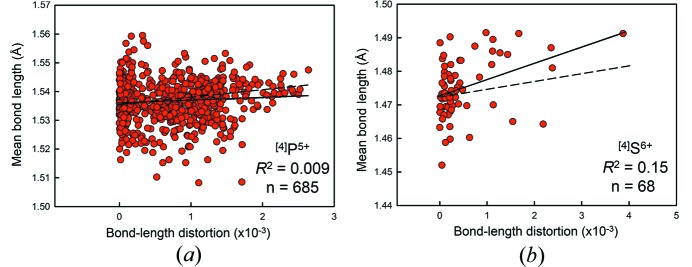
Bond-length distortion plots for (*a*) ^[4]^P^5+^ and (*b*) ^[4]^S^6+^, showing no correlation between bond-length distortion and mean bond length. The dashed line indicates the equation predicted by the distortion theorem.

Fig. 3 ▸ shows the variation in mean bond length as a function of distortion for ^[6]^Zn^2+^, ^[6]^Ti^4+^, ^[6]^V^4+^, ^[6]^Ta^5+^, ^[6]^Mo^6+^, ^[5]^Cu^2+^, ^[6]^Cu^2+^ and ^[6]^Nb^5+^, which are statistically significant at the 99% confidence level and have *R* ^2^ ≥ 0.60. Fig. 4 ▸ shows plots for ^[4]^P^5+^ (*n* = 685, *R* ^2^ = 0.01) and ^[4]^S^6+^ (*n* = 68, *R* ^2^ = 0.15), which despite being statistically significant at the 99% confidence level show very low correlation to bond-length distortion. Fig. 4 ▸(*a*) is a good example of how *R* ^2^ values must not be used to draw conclusions with regard to correlation between mean bond length and other variables, as is often the case; whereas an *R* ^2^ value of 0.01 may seem to indicate that mean bond length is insignificantly correlated to bond-length distortion for ^[4]^P^5+^, the *p*-value of 8.5 × 10^−13^ clearly indicates the contrary, whereby the null hypothesis that the slope of the correlation is zero is rejected at the 99% confidence level. In this case, extreme variation above and below the trend line due to other factors leads to an extremely low *R* ^2^ value, and should not be confused with the significance of the correlation with distortion. Thus this example stresses that the important parameter in determining a correlation is the *p*-value obtained from a Student *t*-test, whereas *R* ^2^ measures how much of the variation in the dependent variable is correlated with the independent variable.

The relation between mean bond length and bond-length distortion may be predicted by expressing the bond lengths in equation (1) as bond valences. From the bond-valence model (Brown, 2002 ▸, 2016 ▸), bond valence is related to bond length
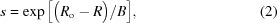
where *s* is the bond valence of a bond of length *R*, and *R*_o_ and *B* are the bond-valence parameters of the ion pair. We may re-arrange equation (2) to
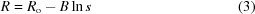
and for mean bond lengths (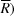 to
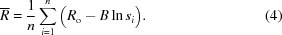
We may then substitute *R* and 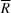 into equation (1), where the sum outside the brackets is taken over the *n* bonds of the polyhedron
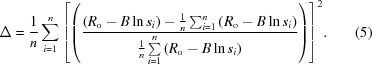
Thus equation (5) gives the predicted distortion value of a coordination polyhedron as a function of bond valence and bond-valence parameters *R*_o_ and *B* for any ion pair. The advantage of using bond valences directly for the purpose of these calculations is that they are easily adjusted to conform with the valence-sum rule, where the sum of the bond valences of an ion is exactly equal to its oxidation state; this is much less straightforward with equation (1). It has been noted that different arrangements of bond valences *s* give slightly different relations between mean bond length and distortion (Urusov, 2003 ▸), and that the value of Δ calculated with equation (5) unescapably depends on the details of the individual bond valences of the polyhedron. However, it is only at extreme values of distortion that the relations for the different modes of distortion become significantly different.

We have calculated the relation between distortion and mean bond length using equation (5) for values of distortion typical of the ion pairs studied. In Figs. 3 ▸ and 4 ▸, the solid line is the fit to the experimental data, and the dashed line is the predicted curve calculated using data points generated from equation (5). Comparison of equation (5) with the observed slopes for the 42 ion configurations that show statistically significant correlations with distortion at the 95% confidence level shows an average difference of 96% in the observed and calculated slopes; this value varies only slightly when using high values for distortion. From the stepwise multiple regression, we know that these deviations do not correlate significantly with variations in the other parameters tested here (mean anion coordination number, electronegativity of the next-nearest-neighbour cations, ionization energy of the next-nearest-neighbour cations). Thus there are two possible explanations: (1) the lack of agreement is due to small sample size [which does not accord with the data for ^[4]^P^5+^, Fig. 4 ▸(*a*)]; (2) there is another significant parameter that has not been identified as yet (discussed below).

We note that on the basis of *R* ^2^ for those significant correlations, mean bond length generally correlates strongly with bond-length distortion for highly distorted configurations and weakly for weakly distorted configurations, in accord with Brown & Shannon (1973 ▸). However, two of the four strongest correlations observed in this study are for weakly distorted configurations: ^[6]^Zn^2+^, *n* = 16, *R* ^2^ = 0.76 and ^[6]^Ta^5+^, *n* = 35, *R* ^2^ = 0.73. ZnO_6_ was one of the six octahedrally coordinated configurations studied by Brown & Shannon (1973 ▸), and they obtained *R* ^2^ = 0.41, also for *n* = 16; this difference in *R* ^2^ value is an example of how sample sizes lower than ∼ 100 coordination polyhedra may show significant variability in *R* ^2^ values (see *The effect of sample size* [Sec sec5]).

### 7.2. Mean coordination number of the bonded anions   

**Figure 5:**
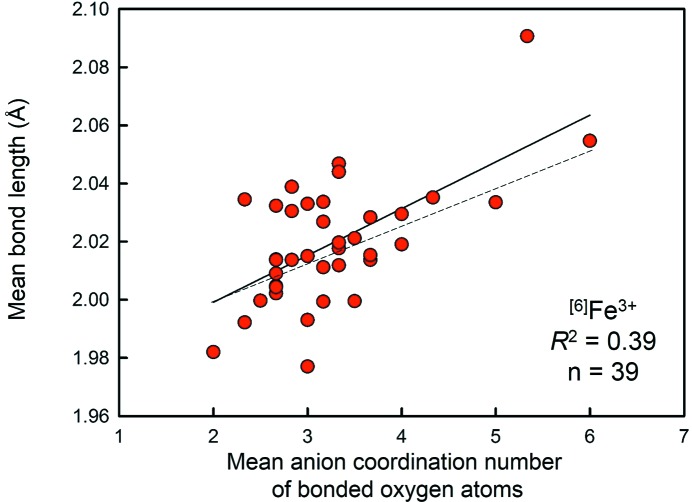
Mean bond length as a function of the mean anion coordination number of the bonded oxygen atoms for Fe^3+^O_6_.

There are 14 of 55 correlations that are statistically significant at the 95% confidence level for mean coordination number of the bonded anions, with a mean *R* ^2^ value of 0.19 (weighted by the number of coordination polyhedra, *R* ^2^ = 0.04) obtained for an average sample size of 68 polyhedra. Fig. 5 ▸ shows data for the strongest correlation, Fe^3+^O_6_, with *n* = 39 and *R* ^2^ = 0.39, where the dashed line gives the correlation expected by summing the radii given by Shannon (1976 ▸).

The combination of (1) the small number of statistically significant correlations, (2) the mean *R* ^2^ value of those that are and (3) concerns about sample size, brings into question the general significance of the mean coordination number of the bonded anions as a causal factor of mean bond-length variation. However, this concept is embedded in the scientific literature (perhaps epitomized by a citation count of ∼ 40 000 for Shannon, 1976 ▸) and requires further consideration. Thus it is necessary for us to consider the original work on which this idea is based.

Brown & Gibbs (1969 ▸) considered the variation of 〈^[4]^Si^4+^—O〉 as a function of the mean coordination number of O^2−^ in 46 minerals; linear regression gave *R* ^2^ = 0.60, but the correlation was not tested for statistical significance. The study of Brown and Gibbs focused specifically on minerals and explicitly excluded Na silicates (and also all other alkali-metal silicates) ‘because of the highly electropositive nature of Na and the concomitant development of strong *d*–*p* π-bonding’. However, the subsequent development of empirical ionic radii (Shannon & Prewitt, 1969 ▸) placed no such restrictions on the use of these radii, which are widely used for all inorganic structures, including alkali-metal oxides. The question arises as to whether the correlation given by Brown & Gibbs (1969 ▸), which Shannon & Prewitt (1969 ▸) describe as pivotal in deriving anion radii as a function of coordination number, is generally applicable.

**Figure 6:**
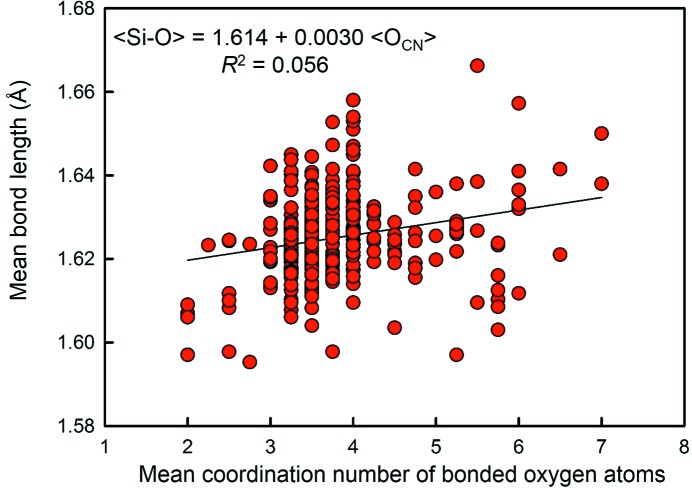
Mean Si—O distance *versus* mean coordination number of the bonded oxygen atoms for 334 SiO_4_ coordination polyhedra.

The plot of 〈Si—O〉 distance *versus* mean anion coordination number for our sample of 334 〈Si—O〉 distances is shown in Fig. 6 ▸; *R* ^2^ is 0.056 and the resulting regression equation is 〈Si—O〉 = 1.614 (3) + 0.0030 (7) 〈O_CN_〉. The resulting *p*-value of 0.00119 is significant at the 99% confidence level, and hence 〈Si—O〉 has a significant correlation with mean anion coordination number even though it involves only a small amount of the total variation in 〈Si—O〉 as indicated by the value of *R* ^2^. The corresponding equation of Brown & Gibbs (1969 ▸), for *n* = 46, is 〈Si—O〉 = 1.579 + 0.015 〈O_CN_〉, with a slope five times as steep as that in Fig. 6 ▸.

**Figure 7:**
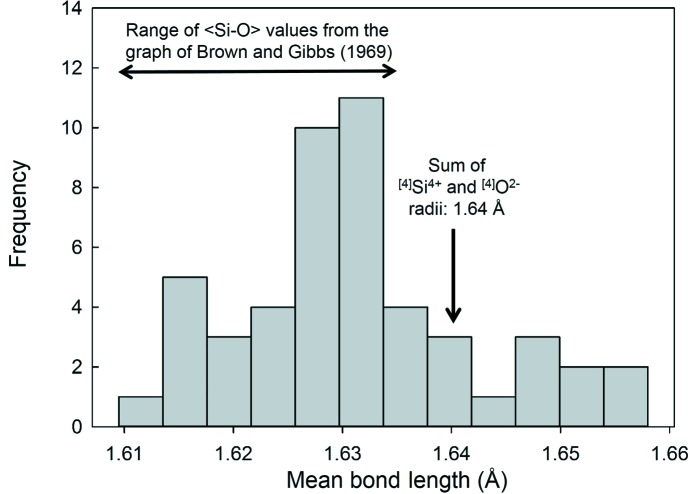
Distribution of mean Si—O distances for structures with a mean coordination number for O^2−^ of [4]. The range of 〈Si—O〉 values taken from the trend line on the graph of Brown & Gibbs (1969 ▸) and the sum of the ^[4]^Si^4+^ and ^[4]^O^2−^ radii from Shannon (1976 ▸) are shown.

Next, we extracted all 〈Si—O〉 values for structures with a mean coordination number for O^2−^ of [4] from our data set of 334 coordination polyhedra, resulting in 49 mean distances. Fig. 7 ▸ shows a histogram of these values, together with the range of 〈Si—O〉 values taken from the trend line on the graph of Brown & Gibbs (1969 ▸) and the sum of the ^[4]^Si^4+^ and ^[4]^O^2−^ radii from Shannon (1976 ▸). The total variation in 〈Si—O〉 distances we observe here for a mean anion coordination of [4] has a range twice that of the distances used to establish the correlation of 〈Si—O〉 distance to mean anion coordination number of Brown & Gibbs (1969 ▸) (whose mean anion coordinations ranged from [2] to [4]). According to the idea that a mean bond length may be predicted from the sum of the coordination-dependent ionic radii for both cation and anion, all data in Fig. 7 ▸ should fall exactly at the sum of the ionic radii for ^[4]^Si^4+^ and ^[4]^O^2−^: 1.640 Å. As is obvious from Fig. 7 ▸, this is clearly not the case; we observe nearly as much variation in mean bond length for a mean anion coordination number of [4] (∼ 1.61–1.66 Å) as there is for all data (∼ 1.59–1.66 Å), calling into contention the idea that the principal cause of variation in mean bond length for a specific ion configuration (*e.g.* ^[4]^Si^4+^ bonded to O^2−^) is variation in the mean coordination number of the bonded anions.

#### 7.2.1. Prediction of mean bond length   

**Figure 8:**
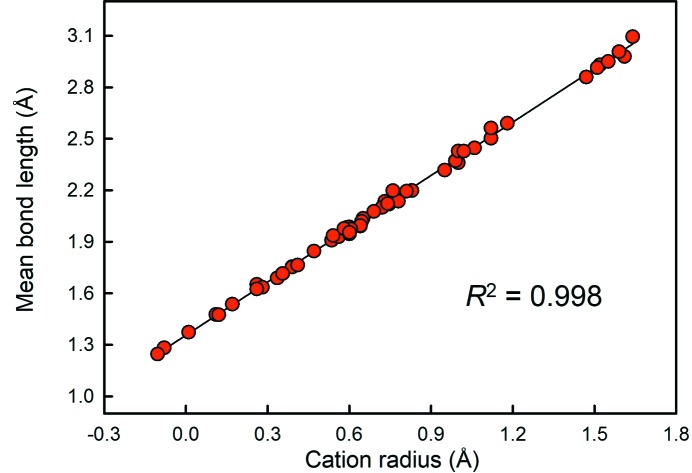
Mean bond length as a function of Shannon (1976 ▸) cation ionic radius for the 55 ion configurations considered in this work.

**Figure 9:**
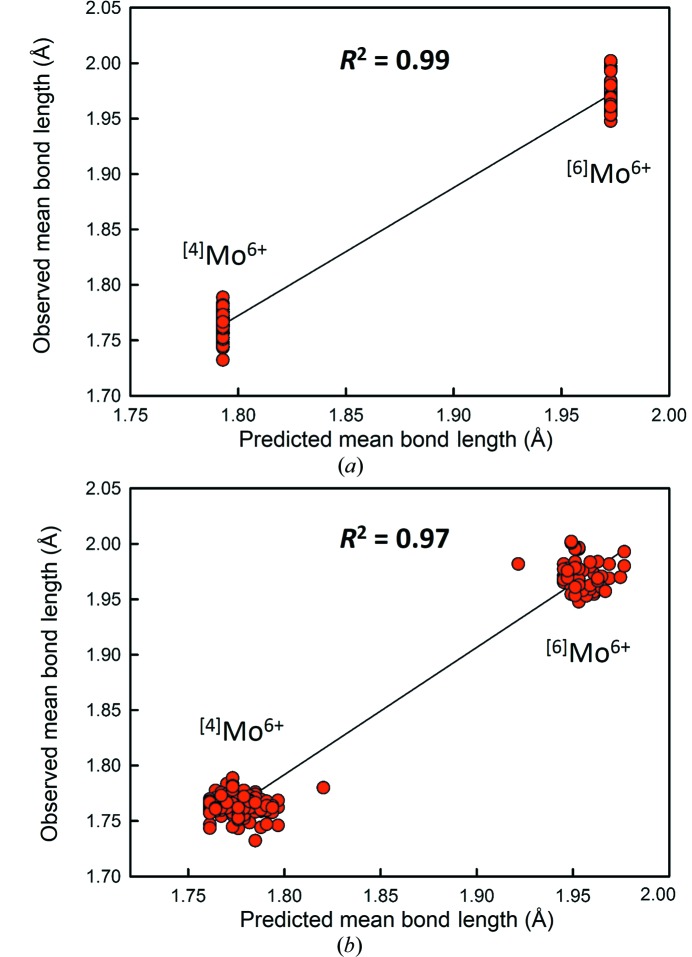
Prediction of mean bond length for Mo^6+^O_6_ octahedra (*n* = 230) from the addition of Shannon (1976 ▸) cation radius and (*a*) 1.38 Å for the ionic radius of oxygen, independent of coordination number, and (*b*) Shannon (1976 ▸) ionic radius of oxygen, dependent of coordination number.

In Fig. 8 ▸, we correlate the mean bond lengths of the 55 ion configurations studied here to the cation radii derived by Shannon (1976 ▸), resulting in a *R* ^2^ value of 0.998. This being the case, we may test the hypothesis that assigning different radii to different anion coordinations of O^2−^ results in the prediction of more accurate mean bond lengths. We have tested this hypothesis for 11 ions and 1703 coordination polyhedra for which the mean coordination number of the bonded oxygen atoms are known. We generated two sets of data: (1) Shannon (1976 ▸) cation radius + 1.38 Å, and (2) Shannon (1976 ▸) cation + anion radius, *i.e.* both function of coordination number. We used 1.38 Å for the fixed radius of O^2−^ as it is the mean observed ionic radius for that sample. Sums (1) and (2) were then compared to the observed mean bond lengths, and the resulting *R* ^2^ values are given in Table 3 ▸. Fig. 9 ▸ shows the match for Mo^6+^ in coordination numbers 4 and 6.

We list *p*-values in Table 3 ▸ with regard to the probability that sets (1) and (2) are identical. Thus, we find that for nine of the 11 ions considered, assigning O^2−^ a radii dependent on its mean coordination number leads to results statistically identical at the 95% confidence level to those obtained by assigning O^2−^ a fixed radius. For the other two ions (B^3+^ and Mo^6+^), we find that using a variable anion coordination number leads to significantly worse results (at the 95% confidence level) than those obtained using a fixed radius for O^2−^.

Whereas Student *t*-tests show that mean bond lengths are significantly correlated with the mean coordination number of the bonded anions for some ion configurations, the *R^2^* values associated with these correlations are very low (Table 2 ▸), indicating that even when statistically significant, the contribution of variable bonded-anion coordination number to the total variation of mean bond length is very small (of the general order of 5%).

In light of the above results, we conclude that the use of current anion radii for different mean bonded-anion coordination numbers is not justified.

### 7.3. Electronegativity of the next-nearest-neighbour cations   

We treated electronegativity in the same way as Hawthorne & Faggiani (1979 ▸), who calculated the mean electronegativity of the next-nearest-neighbour cations (as opposed to taking an average of the electronegativity of all cations in the structure). We tested four scales of electronegativity: Allred & Rochow (1958 ▸), Pauling (1960 ▸), Zhang (1982 ▸) and Allen (1989 ▸), and elected to use the scale of Pauling (1960 ▸) as it gave marginally better results.

Of the eight statistically significant correlations (95% confidence level) for mean electronegativity of the next-nearest-neighbour cations, a mean *R* ^2^ value of 0.00 (weighted, 0.00) is obtained for an average sample size of 125 polyhedra. Removing the potentially overwhelming effect of ^[4]^P^5+^ leads to no change in *R* ^2^ values. Thus it seems that Baur (1971 ▸) was correct in dismissing this potential factor as of no significance (discussing SiO_4_) and we do not consider it further.

### 7.4. Ionization energy of the next-nearest-neighbour cations   

There has been no previous attempt to correlate the mean ionization energy of the next-nearest-neighbour cations to mean bond length.

Of the 13 statistically significant correlations (95% confidence level) for mean coordination number of the bonded anions, a mean *R* ^2^ value of −0.12 (weighted, −0.03) is obtained for an average sample size of 114 polyhedra. Using a 99% confidence level leads to the same *R* ^2^ values. Although we observe correlations that are stronger than those to mean electronegativity of the next-nearest-neighbour cations, there are no grounds to qualify these results as significant; the correlation is not observed for 42 of the 55 ion configurations studied, and those that are correlated give low *R* ^2^ values on a scale that is questionable with regard to sample size (as discussed above). A similar attempt to correlate Lewis acid strength to mean bond length gave very similar results, as Lewis acid strength is highly correlated with ionization energy (*R* ^2^ = 0.90 for 135 cations; Gagné & Hawthorne, 2017*c* ▸). These results give no new insight and are not reported.

### 7.5. The effect of structure type   

The prediction of bond lengths in solids has been proposed by drawing a parallel between crystal structures and electrical networks, and by solving the equivalent of Kirchhoff’s circuit laws for the network of chemical bonds to obtain *a priori* bond valences (Mackay & Finney, 1973 ▸; Brown, 1977 ▸, 1981 ▸, 1987 ▸; Rutherford, 1990 ▸, 1998 ▸; O’Keeffe, 1990 ▸). These equivalent rules for chemical-bond networks are called the valence-sum rule and the equal-valence rule by Brown (1977 ▸, 2002 ▸, 2016 ▸). The *a priori* bond valences calculated by this method are intrinsic to every crystal structure. By assigning specific ions to the sites of the structure, *a priori* bond lengths may be calculated using the appropriate bond-valence parameters, giving the length that each bond in the structure would ideally adopt. Despite the number of times this method has been proposed by different authors, there has been little follow-up work with regard to its application.

**Figure 10:**
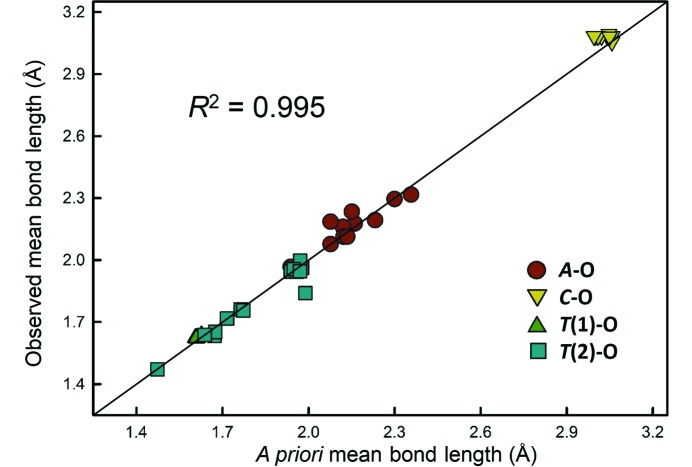
Observed mean bond length *versus a priori* mean bond length for 14 milarite-group minerals for which a reliable structure refinement and chemical analysis are available. Shown for fully occupied sites; plotted line is for a 1:1 correlation.

With regard to variations in *individual* bond lengths, Gagné & Hawthorne (2016*b* ▸) showed a very high correlation between observed and *a priori* bond lengths for the compositionally rich milarite-group minerals (*n* = 111 bonds, *R* ^2^ = 0.99), showing that adherence to the variety of *a priori* bond lengths calculated for the individual sites of a crystal structure is a primary candidate as the principal underlying cause of *individual* bond-length variation in crystals. In Fig. 10 ▸, we show that this correlation is also present for the *mean* bond lengths, where the solid line denotes a 1:1 relation). Thus within a structure type, the *a priori* bond lengths are highly correlated with the observed mean bond lengths. The same behaviour was shown by Bosi & Lucchesi (2007 ▸) who correlated *a priori* to observed mean bond lengths for sites in the tourmaline structure.

**Figure 11:**
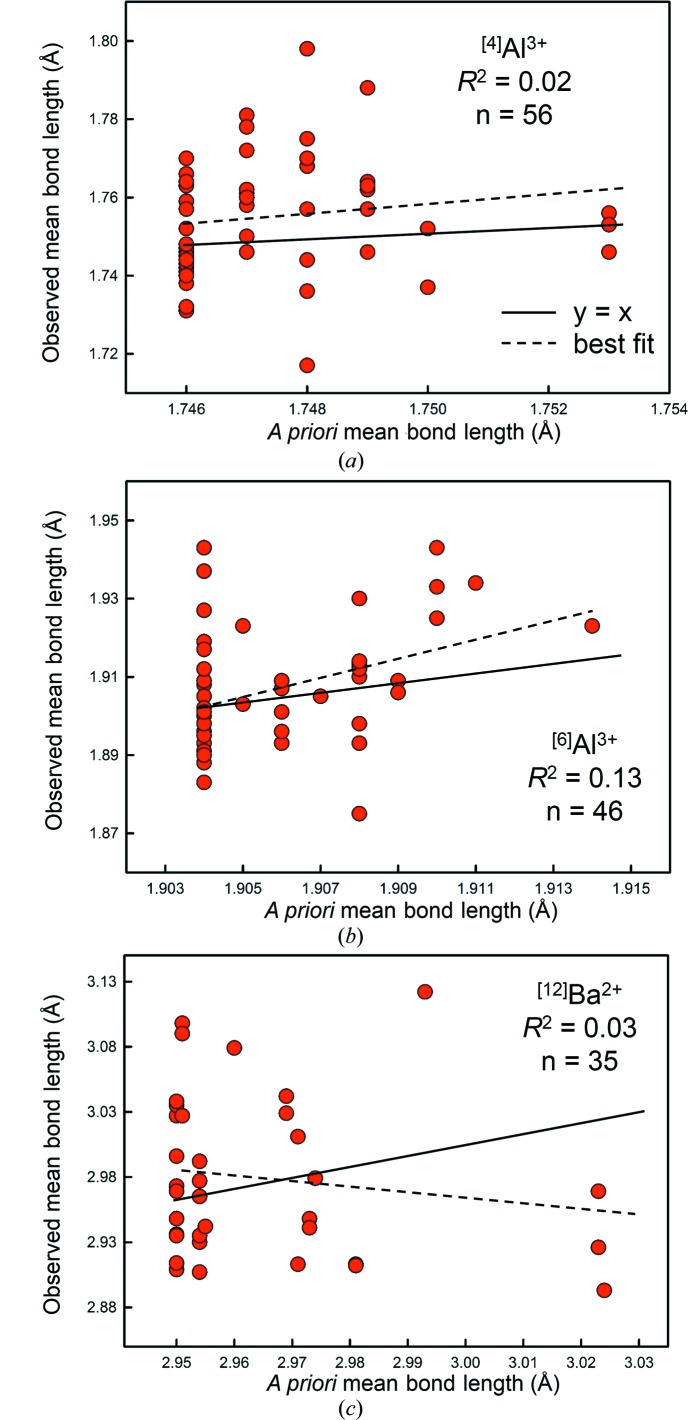
Observed mean bond length *versus a priori* mean bond length for (*a*) ^[4]^Al^3+^, (*b*) ^[6]^Al^3+^ and (*c*) ^[12]^Ba^2+^, from 27, 26 and 25 structure refinements, respectively. The solid line is for a 1:1 correlation; dashed line represents the best-fit equation. These plots show the inability of the *a priori* bond lengths to reproduce observed mean bond lengths to any significant extent between structure types.

To investigate the effect of different structure types on the correlation of *a priori* mean bond lengths with observed mean bond lengths, we solved for the *a priori* bond valences of 27 structures containing ^[4]^Al^3+^ (56 polyhedra), 26 structures containing ^[6]^Al^3+^ (46 polyhedra), and 25 structures containing ^[12]^Ba^2+^ (37 polyhedra), and calculated the corresponding *a priori* bond lengths and mean bond lengths using the bond-valence parameters of Gagné & Hawthorne (2015 ▸) (see Table S1). The variation of observed mean bond length with *a priori* mean bond length is shown in Fig. 11 ▸, where the solid lines are for *y* = *x*, and the dashed lines are the least-squares fit. The correlation is statistically significant at the 95% confidence level for ^[6]^Al^3+^ only [*p*-value 0.015, with *R* ^2^ = 0.13; Fig. 11 ▸(*a*)]. The lack of correlation for ^[4]^Al^3+^ and ^[12]^Ba^2+^ structures [Figs. 11(*b*) and 11(*c*)] and the very small *R* ^2^ for the correlation involving the ^[6]^Al^3+^ structures indicates that *a priori* bond lengths do not reproduce observed mean bond lengths to any significant extent between structure types.

#### 7.5.1. Structural strain: a measure of the effect of structure type on mean bond lengths   

It is apparent from this empirical evidence that structure type is a major control on the variation in mean bond length for specific ion configurations, and that the magnitude of this effect goes well beyond that of the other variables analyzed in this work.

The following issues arise: (1) how does one describe this effect in a manner that relates to the bond topology of a crystal structure and (2) how does one reduce this description to a simple scalar quantity that one can correlate with variations in mean bond length?

Any finite graph can be embedded in three-dimensional Euclidean space. This embedding maintains the connectivity of the graph, but does not necessarily maintain any metric aspects that one wishes to associate with the edges of that graph. This issue is of particular importance with regard to the principal axioms of bond-valence theory, the valence-sum rule and the loop rule (Brown, 2002 ▸, 2016 ▸). As noted above, *a priori* bond valences can be calculated by applying these axioms to the topology of the bond network with specific ions at specific vertices and the corresponding *a priori* bond lengths can be calculated from bond-valence parameters.

The inability of a structure to adopt its a *priori* bond lengths causes stress that produces strain in the structure. Brown (2014 ▸) defined steric constraints as ‘those that arise when a bond network cannot be mapped into three-dimensional space without straining the *a priori* bond lengths calculated with the network equations’, whereby some bonds may have to be stretched and others compressed for the structure to ‘fit’ into three-dimensional space. To this definition, Brown (2016 ▸) adds the constraints of space-group symmetry, because as well as fitting into three-dimensional space, the structure is also constrained to obey the symmetry properties of its space group.

In a theoretical study of mean bond lengths using the bond-valence model, Bosi (2014 ▸) states that observed mean bond lengths result from the addition of the mean bond length for an undistorted polyhedron and a correction term for distortion. He then breaks down the distortion term into several terms: distortion caused by (*a*) the topology of the structure, *i.e.* the non-equivalence of bonds, (*b*) isotropic steric strain, (*c*) anisotropic steric strain, and (*d*) anisotropic electronic strain. Bosi concludes that the difference between an observed and a theoretical coordination polyhedron is caused by the occurrence of strain.

Thus the degree of fit between an *a priori* structure and an observed structure has been designated as *strain*, and various definitions have been proposed for this. The following was proposed by Brown (2014 ▸):
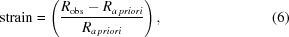
where the magnitude of the strain of individual bonds results from the proportional difference between the observed bond length *R*_obs_ and its corresponding *a priori* bond length, 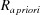. More comprehensive ways in which structural strain can be measured have been proposed with regard to the bond-valence model with the calculation of the Global Instability Index (GII; Salinas-Sanchez *et al.* 1992 ▸), and the Bond Strain Index (BSI; Preiser *et al.* 1999 ▸). The GII evaluates the difference between the bond-valence sums at the sites of the structure compared to their ideal values by calculating the root-mean-square deviation of the bond-valence sums from their atomic valences, averaged over all atoms in the formula unit
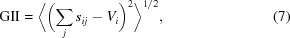
where *s_ij_* is the observed bond-valence of ion *i* with coordination number *j*. The BSI is defined as the root-mean-square deviation between the *a priori* and observed bond valences, averaged over all bonds in the formula unit
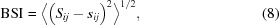
where 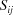 is the *a priori* bond valence.

The inability of a structure to attain its *a priori* bond lengths within the constraints of its space-group symmetry will lead to adjustments in the structure whereby the (mean) bond lengths adjust from their set of ideal values to some set of compromise values that do conform to the space-group symmetry of the crystal. Strain as expressed by the BSI is a good expression of that compromise.

It is probable that the way forward in understanding variations in mean bond lengths in crystals will involve:

(1) Calculating the *a priori* bond valences and bond lengths for a wide variety of structure types for each ion configuration of interest.

(2) Calculating the bond strain index (BSI) for these structures.

(3) Correlating bond topology, BSI and space-group symmetry with variations in mean bond lengths.

For point (3), we note that some of these factors have partially and implicitly been correlated to mean bond length *via* the bond-length distortion index. For example, bond-length distortion as represented in equation (1) is inclusive of the effect of structural distortion for given bond topologies (distortion caused by the fact that different bond topologies have different *a priori* bond valences, *i.e.* not always the Pauling bond strength, which inherently creates some variation in mean bond lengths). It could also be argued that space-group symmetry is implicitly embedded into the bond-length distortion index, as the physical distortion of the polyhedron changes the symmetry of the structure. However, implicit inclusion of these factors in the distortion index is unsatisfactory for the purpose of demonstrating statistical significance. The extent for which these factors individually contribute to mean bond-length variations, and their underlying mechanism, are unclear; a way to examine these factors explicitly must be devised.

There are many indices that may be used to describe the topology of a crystal structure (*e.g.* the number of topologically independent bonds, and their multiplicity) and the symmetry properties of the corresponding space group (*e.g*. the number of symmetry operations), and what variables can be used to quantitatively represent bond topology and space-group symmetry is not immediately clear. It seems probable that their proper examination, as part of work on points (1) to (3), will result in a clarification of the reasons underlying the overwhelming effect that structure type has on mean bond length.

## 8. Summary and conclusion   

(1) Following a review of previous work on the variation of mean bond length in oxide and oxysalt crystals, we use 55 cation configurations bonded to O^2−^ to analyze the relation between mean bond lengths and (*a*) bond-length distortion, (*b*) mean coordination number of the oxygen atoms bonded to the cation, (*c*) mean electronegativity of the next-nearest-neighbour cations, and (*d*) mean ionization energy of the cations bonded to the oxygen atoms of the coordination polyhedron *via* stepwise multiple regression analysis at the 95% confidence level.

(2) Of the 55 ion configurations examined, 42 show a correlation between mean bond length and bond-length distortion significant at the 95% confidence level. However, a mean *R* ^2^ of 0.35 indicates that mean bond length must correlate with other factors hitherto not identified.

(3) We find that previously published correlations between mean bond length and mean coordination number of the bonded anions are not of general applicability to inorganic oxide and oxysalt structures.

(4) Compared to assigning O^2−^ a fixed radius, use of currently accepted anion-coordination-dependent radii for O^2−^ in the prediction of mean bond lengths leads to statistically identical results at the 95% confidence level for nine of 11 ions tested, and less accurate predictions for the other two ions using the anion-coordination-dependent radii for O^2−^.

(5) Points (3) and (4) indicate that the currently accepted ionic radii for O^2−^ in different coordinations are not justified by the experimental data.

(6) We find no correlation between mean bond length and the mean electronegativity and mean ionization energy of the cations bonded to the oxygen atoms of the coordination polyhedron.

(7) Calculation of *a priori* bond lengths for many ion configurations in a single structure-type leads to linear relations between *a priori* and observed mean bond lengths. However, calculation of *a priori* bond lengths for a single ion configuration in many different structure-types leads to negligible correlation between *a priori* and observed mean bond lengths across structure types.

(8) We suggest that the wide variation in mean bond length for a single ion configuration is a result of the inability of a structure to attain its ideal (*a priori*) bond lengths within the constraints of its space-group symmetry. The structure adjusts from its set of ideal bond lengths (and corresponding mean bond lengths) to some set of ‘compromise’ bond lengths that do conform to the space-group symmetry of the crystal, and the Bond Strain Index (Preiser *et al.*, 1999 ▸) is an expression of the magnitude of that compromise.

It is apparent that future work on understanding variations in mean bond length in crystals should be directed toward the elucidation of the stress created from the mismatch between *a priori* and observed bond valences in crystal structures. This will entail the calculation of *a priori* bond valences and bond lengths in a wide variety of structure types, to be compared with observed bond valences and bond lengths *via* various strain indices. In turn, it will be necessary to derive simple descriptors for (1) bond topology, and (2) space-group symmetry that may be examined for correlation with mean bond length.

## Supplementary Material

Table S1. DOI: 10.1107/S2052520617014548/bm5096sup1.pdf

## Figures and Tables

**Table 1:** Evolution of *p*-values, *R* ^2^ and adjusted *R* ^2^ as variables are added to the stepwise multiple-regression analysis for NaO_6_ (*n* = 112 coordination polyhedra) 〈*IE*〉 is the mean ionization energy and 〈χ〉 is the mean electronegativity. Numbers in italics indicate lowest *p*-value.

	*p*-value					
Step	Distortion	〈χ〉 of next-nearest-neighbour cation	〈*IE*〉 of next-nearest-neighbour cation	Bonded anion 〈CN〉	*R* ^2^	Adjusted *R* ^2^
1	*5.4 × 10^−8^*	1.5 × 10^−3^	0.26	1.9 × 10^−3^	0.24	
2		*7.9 × 10^−3^*	0.41	8.0 × 10^−3^	0.28	0.27
3			*0.045*	0.24	0.31	0.29
4				1.6 × 10^−3^	0.37	0.35

**Table 2:** Coefficients of determination *R* ^2^ of the individual correlations to mean bond length for samples statistically significant at 95% confidence intervals (normal font) and at 99% confidence intervals (bold font) from stepwise multiple-regression analysis, and adjusted *R* ^2^ values for ion configurations for which two or more potential factors are statistically significant at 95% confidence intervals 〈*IE*〉 is the mean ionization energy and 〈χ〉 is the mean electronegativity. Dashes indicate lack of statistical significance.

	Sample size	Distortion	Bonded anion 〈CN〉	〈χ〉 of next-nearest-neighbour cation	〈*IE*〉 of next-nearest-neighbour cation	Adjusted *R* ^2^
^[6]^Ti^4+^	57	**0.75**	**0.21**	–	–	0.81
^[5]^Cu^2+^	22	**0.65**	**0.19**	–	–	0.77
^[6]^Ta^5+^	35	**0.73**	–	–	**−0.01**	0.77
^[6]^Zn^2+^	16	**0.76**	–	–	–	
^[6]^V^4+^	29	**0.74**	–	–	–	
^[6]^Cu^2+^	29	**0.63**	**0.24**	–	–	0.72
^[6]^Nb^5+^	38	**0.60**	**0.17**	–	–	0.71
^[6]^Mo^6+^	59	**0.68**	–	–	–	
^[6]^Co^2+^	24	**0.51**	–	–	**−0.14**	0.66
^[6]^Mn^2+^	43	**0.57**	–	–	−0.03	0.62
^[6]^Te^6+^	21	**0.28**	–	** 0.10**	**−0.04**	0.59
^[6]^Fe^3+^	39	**0.34**	**0.39**	–	–	0.59
^[6]^V^5+^	20	**0.50**	–	0.16	–	0.56
^[12]^Ba^2+^	47	**0.55**	–	–	–	
^[6]^W^6+^	35	**0.50**	–	–	–	
^[4]^Ga^3+^	27	–	0.29	**−0.17**	**−0.33**	0.48
^[6]^Sb^5+^	19	**0.45**	–	–	–	
^[6]^Cd^2+^	26	**0.39**	–	–	–	
^[9]^Ba^2+^	54	**0.39**	–	–	–	
^[7]^Ca^2+^	30	**0.28**	–	–	−0.10	0.38
^[4]^Se^6+^	21	**0.35**	–	–	–	
^[4]^B^3+^	148	**0.33**	**0.05**	–	–	0.35
^[6]^Na^+^	112	**0.24**	**0.08**	** 0.09**	** 0.01**	0.35
^[6]^Al^3+^	49	**0.23**	**0.15**	–	–	0.34
^[4]^Be^2+^	29	0.22	0.28	–	–	0.34
^[9]^K^+^	50	**0.33**	–	–	–	
^[8]^Ca^2+^	53	**0.32**	–	–	–	
^[5]^Na^+^	25	**0.32**	–	–	–	
^[6]^Mg^2+^	45	**0.17**	**0.13**	–	–	0.32
^[6]^Li^+^	26	0.15	–	−0.21	–	0.30
^[4]^Zn^2+^	32	–	–	–	**−0.29**	
^[6]^Fe^2+^	28	**0.27**	–	–	–	
^[6]^Ca^2+^	23	–	–	–	−0.26	
^[6]^Ni^2+^	52	–	**0.22**	0.00	–	0.26
^[10]^Ba^2+^	56	**0.02**	–	–	**−0.17**	0.26
^[4]^Cr^6+^	28	**0.24**	–	–	–	
^[7]^Na^+^	31	0.21	–	–	–	
^[4]^Li^+^	88	**0.19**	–	–	–	
^[4]^Al^3+^	58	–	**0.17**	–	–	
^[10]^K^+^	40	**0.17**	–	–	–	
^[8]^K^+^	26	0.16	–	–	–	
^[4]^S^6+^	68	**0.15**	–	–	–	
^[7]^U^6+^	69	**0.15**	–	–	–	
^[4]^As^5+^	59	–	–	0.02	**−0.09**	0.15
^[4]^P^5+^	685	**0.01**	–	**−0.01**	**−0.07**	0.14
^[3]^B^3+^	237	–	**0.10**	–	–	
^[4]^Mo^6+^	171	**0.09**	–	–	–	
^[4]^Si^4+^	335	–	–	–	**−0.08**	
^[3]^C^4+^	67	−0.07	–	–	–	
^[4]^V^5+^	96	0.06	–	–	–	
^[3]^N^5+^	37	–	–	–	–	
^[4]^Na^+^	32	–	–	–	–	
^[8]^Na^+^	42	–	–	–	–	
^[12]^K^+^	31	–	–	–	–	
^[4]^Ge^4+^	64	–	–	–	–	
Mean *R* ^2^		0.35	0.19	0.00	−0.12	
Weighted mean *R* ^2^		0.16	0.04	0.00	−0.03	

**Table 3:** Prediction of mean bond length with and without discrimination for anion coordination number

	*n*	Cation + anion radius (Å)	Cation radius + 1.38 (Å)	*p*-value
Al^3+^	107	0.96	0.96	0.44
B^3+^	385	0.96	0.97	**0.00**
Ba^2+^	157	0.41	0.41	0.72
Ca^2+^	106	0.68	0.69	0.38
Cu^2+^	51	0.80	0.75	0.19
K^+^	147	0.42	0.46	0.59
Li^+^	114	0.80	0.80	0.24
Mo^6+^	230	0.97	0.99	**0.02**
Na^+^	242	0.50	0.55	0.55
V^5+^	116	0.96	0.98	0.13
Zn^2+^	48	0.90	0.92	0.42
Mean		0.76	0.77	0.33
