# Supplementary material for: Mean bond-length variations in crystals for ions bonded to oxygen
Source: Acta Crystallogr B Struct Sci Cryst Eng Mater. 2017 Nov 28;73(Pt 6):1019–31. doi: 10.1107/S2052520617014548 (PMC5744403; doi:10.1107/S2052520617014548)
Supplement: Supplementary file 1 [file b-73-01019-sup1.pdf]

STRUCTURAL SCIENCE
CRYSTAL ENGINEERING
MATERIALS

Volume 73 (2017)

Supporting information for article:

Mean bond-length variations in crystals for ions bonded to oxygen

Olivier Charles Gagné and Frank Christopher Hawthorne

Table S1 *A priori* mean bond-lengths of [4]Al^{3+}, [6]Al^{3+} and [12]Ba^{2+} polyhedra

ICSD code	CP #	*A priori* <BL> (Å)	Observed <BL> (Å)	Difference (Å)
[4]Al^{3+}				
29443	1	1.746	1.742	-0.004
33832	1	1.746	1.746	0.000
40530	1	1.748	1.736	0.012
	2	1.746	1.731	0.015
51754	1	1.749	1.757	-0.008
62317	1	1.746	1.759	-0.013
	2	1.746	1.741	0.005
	4	1.746	1.766	-0.019
62490	1	1.747	1.761	-0.014
62615	1	1.746	1.763	-0.017
65004	1	1.746	1.740	0.006
67055	1	1.746	1.770	-0.024
71114	4	1.747	1.750	-0.002
71602	1	1.746	1.747	-0.001
79558	1	1.747	1.781	-0.034
	2	1.747	1.750	-0.003
	3	1.748	1.775	-0.027
	4	1.749	1.788	-0.038
	5	1.748	1.757	-0.010
	6	1.748	1.798	-0.051
	7	1.747	1.746	0.001
	8	1.749	1.788	-0.039
	9	1.748	1.770	-0.022
	10	1.748	1.717	0.031
	11	1.747	1.778	-0.031

	12	1.748	1.744	0.004
85200	2	1.749	1.762	-0.013
91551	1	1.746	1.752	-0.005
95367	1	1.747	1.746	0.001
	2	1.746	1.738	0.008
	3	1.746	1.745	0.001
	4	1.746	1.743	0.003
95408	9	1.748	1.768	-0.020
	10	1.748	1.770	-0.022
95535	1	1.747	1.762	-0.015
	2	1.749	1.764	-0.015
	3	1.749	1.763	-0.014
	4	1.747	1.772	-0.025
	5	1.750	1.752	-0.002
	6	1.747	1.758	-0.011
150769	1	1.746	1.764	-0.018
	2	1.749	1.746	0.003
	3	1.747	1.760	-0.013
154360	1	1.753	1.756	-0.003
154361	1	1.753	1.753	0.000
154362	1	1.753	1.746	0.007
154363	1	1.746	1.752	-0.005
156656	1	1.746	1.743	0.003
	2	1.746	1.745	0.001
156658	1	1.746	1.744	0.003
	2	1.746	1.740	0.007
158747	1	1.746	1.757	-0.011
	2	1.750	1.737	0.013
280977	1	1.746	1.744	0.003
	2	1.746	1.748	-0.001

414553	1	1.746	1.732	0.015
$^{[6]}Al^{3+}$				
20231	1	1.904	1.900	0.004
	2	1.904	1.895	0.009
	3	1.904	1.908	-0.004
	4	1.904	1.909	-0.004
	5	1.904	1.905	0.000
34803	1	1.908	1.910	-0.002
39606	1	1.906	1.907	-0.002
39827	1	1.904	1.893	0.011
50618	1	1.904	1.888	0.016
50626	1	1.904	1.927	-0.023
62615	2	1.904	1.919	-0.015
71114	1	1.908	1.898	0.011
75366	1	1.904	1.891	0.013
83027	1	1.906	1.901	0.005
83450	1	1.904	1.902	0.002
	2	1.904	1.912	-0.007
	3	1.904	1.917	-0.013
	4	1.904	1.896	0.009
85200	1	1.908	1.913	-0.005
95408	1	1.908	1.893	0.015
	2	1.910	1.925	-0.016
	3	1.906	1.893	0.013
	4	1.914	1.923	-0.008
	5	1.906	1.909	-0.003
	6	1.910	1.933	-0.023
	7	1.909	1.909	0.000

	8	1.911	1.934	-0.024
	11	1.905	1.903	0.002
	12	1.907	1.905	0.002
96685	1	1.904	1.895	0.009
99419	1	1.906	1.909	-0.004
100831	1	1.904	1.901	0.003
157730	1	1.908	1.930	-0.022
159977	1	1.908	1.912	-0.004
	2	1.910	1.943	-0.034
160892	1	1.909	1.906	0.003
	2	1.908	1.914	-0.006
165840	1	1.904	1.898	0.006
172780	1	1.905	1.923	-0.018
	2	1.906	1.896	0.010
172781	1	1.904	1.943	-0.039
	2	1.904	1.937	-0.032
240475	1	1.904	1.901	0.003
280947	1	1.904	1.883	0.021
281210	1	1.904	1.890	0.015
414553	2	1.908	1.875	0.033
$^{[12]}Ba^{2+}$				
33804	1	2.954	2.907	0.047
	2	2.954	2.977	-0.023
50040	1	2.950	3.027	-0.076
60580	1	2.954	2.930	0.024
60581	1	2.954	2.935	0.019
62965	1	2.954	2.992	-0.038
66408	1	2.950	2.973	-0.023

68612	1	2.950	3.035	-0.085
71602	3	2.951	3.027	-0.076
72295	1	2.954	2.965	-0.011
77499	1	2.950	2.969	-0.019
80591	1	2.971	2.913	0.058
80812	1	2.971	3.011	-0.040
86043	1	3.024	2.893	0.132
86431	1	2.950	2.996	-0.045
93226	1	2.955	2.942	0.013
95527	1	2.950	3.038	-0.087
97524	1	2.969	3.042	-0.073
	3	2.951	3.098	-0.147
	4	2.973	2.948	0.025
97525	1	2.969	3.029	-0.060
	3	2.951	3.090	-0.139
	4	2.973	2.941	0.032
99661	1	2.950	2.948	0.003
	2	2.950	2.936	0.015
170217	2	2.960	3.079	-0.119
172754	1	2.950	2.909	0.042
	2	2.950	2.914	0.036
200876	1	2.950	2.935	0.015
201621	1	2.974	2.979	-0.005
412837	1	3.023	2.926	0.098
	2	2.981	2.913	0.069
	3	3.023	2.969	0.054
	4	2.981	2.912	0.069
416231	2	2.993	3.122	-0.129
